# Investigating Potential Modes of Actions of* Mimusops kummel* Fruit Extract and Solvent Fractions for Their Antidiarrheal Activities in Mice

**DOI:** 10.1155/2017/4103410

**Published:** 2017-05-09

**Authors:** Mulugeta Molla, Negero Gemeda, Solomon M. Abay

**Affiliations:** ^1^School of Pharmacy, College of Health and Medical Sciences, Haramaya University, Haramaya, Ethiopia; ^2^Traditional and Modern Medicine Research Directorate, Ethiopian Public Health Institute, Addis Ababa, Ethiopia; ^3^Department of Pharmacology, School of Medicine, College of Health Sciences, Addis Ababa University, Addis Ababa, Ethiopia

## Abstract

**Background:**

Fruits of* Mimusops kummel* A. DC. (Sapotaceae) are traditionally used for the treatment of diarrhea. The present study aimed at investigating modes of actions of this fruits for antidiarrheal action to guide future drug development process.

**Methods:**

Fractions of chloroform, n-butanol, and water were obtained from 80% methanol extract, which was prepared by maceration. Antidiarrheal activities and the modes of actions were investigated in mice.

**Results:**

In castor oil induced diarrheal model, the extract delayed onset of diarrhea and reduced number and weight of feces at all tested doses significantly. In this model all fractions significantly delayed onset of diarrhea at all tested doses. Charcoal meal test showed that the extract and all the fractions produced a significant antimotility effect at all tested doses. Enteropooling test showed that the extract as well as n-butanol and aqueous fractions at all tested doses produced a significant decline in volume and weight of intestinal contents, whereas chloroform fraction had substantial effect only at high dose.

**Conclusion:**

This study demonstrated that the extract and solvent fractions produced antidiarrheal activities due to dual inhibitory effect, intestinal motility, and fluid secretion, with the aqueous fraction being the most active among fractions in three models.

## 1. Background

Diarrheal illness is a serious public health problem that affects all regions of the world and all ages [1, 2]. Diarrhea is a state of reversal of normal net absorption of water and electrolyte, absorption to secretion [3]. There are many etiologic factors of diarrhea, but infection is the most common cause. Infectious diarrhea occurs because of food and water contamination via the fecal-oral route [4]. The main enteric pathogens include a wide range of bacteria (e.g., diarrheagenic* Escherichia coli*, nontyphoidal* Salmonella*,* Shigella *spp.,* Vibrio cholera*, and* Campylobacter *spp.), virus (e.g.,* rotavirus *group A,* norovirus*,* Sapovirus*,* astrovirus*,* adenovirus*, and* enterovirus*), and enteric parasites (e.g.,* Cryptosporidium *spp.,* Giardia lamblia, Entamoeba histolytica*, and* Blastocystis hominis*) [2].

In addition to fluid and electrolyte replacement, pharmacologic interventions like adsorbents (e.g., attapulgite), antimotility agents (e.g., loperamide), antisecretory agents (e.g., octreotide), and anti-infective agents (e.g., fluoroquinolones) are the conventional options in the treatment of diarrheal diseases [5, 6]. Rotavirus vaccines included in national immunization programs [7] and a vaccine for cholera that could be useful in some settings in all ages have also been available for several years [1]. For the already available tools used to treat or prevent diarrhea, there are product specific issues: safety concerns with the live attenuated vaccines [8] and increasing rate of microbial resistance to the approved anti-infective drugs [9, 10]. For instance, a study in Cambodia showed that 100% of* Shigella* isolates were resistant to trimethoprim/sulfamethoxazole, which is one of the conventional agents to treat diarrhea [11].

Despite the development of vast spectrum of western medicine approaches for the management of diarrhea, several studies show the reliance of people in developing countries on traditional medicine to intervene the sign and symptoms of diarrhea [12, 13]. This practice may be attributed partly due to the inadequacy and inaccessibility of modern health services [14]. In relation to the indigenous practice, ethnobotany studies show that different types of plant materials are traditionally used for the treatment of diarrhea, among which there are fruits of* M. kummel* A. DC. (Sapotaceae) [12, 13, 15].

*M. kummel *is a deciduous small-to medium-sized tree up to 35 m high, containing latex, with bole up to 100 cm in diameter, bark deeply grooved, dark grey, crown dense, ovoid, and young densely red brown pubescent branches. Leaves arranged spirally, more or less in tufts at the ends of branches. Its fruit is an ellipsoid to ovoid berry up to 2.5 cm long, orange-red when being ripe, containing a single large seed [16].* M. kummel* fruits are principally used in Ethiopia for the treatment of diarrhea and amebiasis [15]. Similarly, the seeds are used to treat ascariasis [16].

An experimental study from Kenya showed that organic solvent extracts of* M. kummel* have antimicrobial activity [17], particularly* Pseudomonas aeruginosa *(causing nosocomial diarrhea) [18]. The same study reveals the presence of sesquiterpene lactones, flavonoids, alkaloids, and saponins in the methanol extract of* M. kummel* stem. Another study from Cote d'Ivoire reports antischistosomal activity of hydroethanol extract of* M. kummel* stem bark. Activity of this extract against newly transformed schistosomula was higher than the activity against an adult worm [19].

The prior reports of antibacterial [17] and antiparasitic [19] activities support use of* M. kummel* fruit in the treatment of diarrhea in Ethiopia. This evidence warrants studies on other responsible modes of action (antimotility and antisecretory) for its antidiarrheal activity, which is conducted using animal models that mimic the pathogenesis of diarrheal illness in human being [20, 21]. To this effect, it is necessary to establish scientific evidence for therapeutic use of* M. kummel*, as it may potentially be useful source of new lead compounds to be input for the drug development process or give a clue about the strategies of standardized medicinal plant remedy. The present study, therefore, aimed at evaluating the mode of antidiarrheal activity of the 80% methanol extract of* M. kummel *fruits (80% MeOH-E) and solvent fractions in mice model.

## 2. Materials and Methods

### 2.1. Collection and Preparation of Plant Materials

Fresh ripe fruits of* M. kummel *were collected from Sisa, Dera Woreda, Amhara region, located about 595 km north of the capital Addis Ababa, Ethiopia, in November 2015. Botanical identification and authentication were done by senior botanist (Dr. Getachew Addis) at the Traditional and Modern Medicine Research Directorate, Ethiopian Public Health Institute (EPHI). A voucher specimen (number MM-01) was deposited at the institute's herbarium for future reference. The fruits were initially washed using distilled water to remove dust materials and dried at room temperature under shade for 14 days. The fruits were then chopped into small pieces and ground into coarse powder using a porcelain mortar and pestle. The powder was stored at room temperature in air sealed polythene bags until extraction commenced.

### 2.2. Extraction and Fractionation

#### 2.2.1. Preparation of 80% Methanol Extract

Hydromethanol (80%) extract was prepared by cold maceration technique. Briefly, 500 g of coarse fruit powder in a conical flask was mixed with 2.5 L of 80% methanol. The flask with its contents was sealed and kept for a period of 48 h at room temperature accompanying intermittent shaking using miniorbital shaker (Bibby Scientific Limited, Stone, Staffordshire, UK) revolving at 120 rpm to enhance the efficient extraction. The entire mixture was first filtered through a funnel plunged with muslin cloth two times and then the filtrate was passed through Whatman filter paper (Number 1) (Maidstone, UK). After filtration, the residue was remacerated two times for a total of 96 h in order to obtain a better yield. The marc was pressed and the combined filtrate was then concentrated using a rotary evaporator (Buchi Model R-200, Switzerland) set at 40°C. The concentrate was pooled together and freeze-dried using a lyophilizer (Operan, Korea Vacuum Limited, Korea). It rendered a solid residue of yellowish color which was designated as 80% MeOH-E and stored in an air tight container in deep freezer (−20°C) until being used for further investigation.

#### 2.2.2. Fractionation of Crude Extract

Solvent fractionation of crude extract was carried out using water, chloroform, and n-butanol. Briefly, eighty grams of the crude extract was dissolved in 400 mL of distilled water and this solution was transferred to a separating funnel. An equal volume of chloroform was added to it and was shaken vigorously. The mixture was separated in two layers. The chloroform layer (lower) was then removed. The partition with chloroform was repeated two times. All of the chloroform layers were combined and subjected to evaporation using a rotary evaporator (Buchi Model R-200, Switzerland) set at 40°C to get the chloroform fraction, and then the filtrate was placed in an oven at 45°C for one week to remove the remaining solvent. To the separating funnel containing aqueous layer, 400 mL of n-butanol was added. The upper layer in this case was n-butanol, which was separated and the procedure was repeated two times. The separated n-butanol layers were pooled and concentrated using a rotary evaporator (Buchi model R-200, Switzerland) set at 40°C to obtain the n-butanol fraction, and then the filtrate was placed in an oven at 45°C for two weeks to remove the remaining solvent. The remaining aqueous layer (lower in this case) was concentrated in a lyophilizer (Operan, Korea Vacuum Limited, Korea) to remove water. After drying, the solvent fractions were stored in an air tight container in refrigerator until being used for evaluation of the antidiarrheal activity and phytochemical constituents.

#### 2.2.3. Experimental Animals

Swiss albino mice of either sex, weighing 25–30 g and aged 6–8 weeks, obtained from Animal Breading Unit of EPHI were used for the experiment. Five to eight animals were housed in polyethylene cages having metallic cover and woodchip bedding. They were maintained at a 12 h light-dark cycle, ambient room temperature, and had free access to standard pellet diet and water. The animals, handled according to the guideline for the care and use of experimental animals [22], were acclimatized to laboratory condition—Pharmacology Laboratory of the Traditional and Modern Medicine Research Directorate, EPHI—for one week before being subjected to experimental protocol [23]. Ethical approval for the conduct of the research project was obtained from the Scientific and Ethics Committee of the Department of Pharmacology, School of Medicine, Addis Ababa University.

#### 2.2.4. Animal Grouping and Dosing

In all the three experimental models, animals were randomly assigned by research assistant into groups (a negative control group, three test groups, and a positive control group) comprising of six animals per group. All groups were provided with their respective treatments using oral gavage. The negative control groups received vehicle at a volume of 10 mL/kg body weight (distilled water only for control group in 80% MeOH-E assessment or 2% Tween 80 in distilled water for the control group in chloroform, n-butanol, and aqueous fractions category). The test groups received 100, 200, and 400 mg/kg of the 80% MeOH-E or solvent fractions of* M. kummel* orally, while the positive group received standard drug (loperamide, 3 mg/kg) orally. Loperamide served as a standard drug for castor oil induced diarrhea, small intestine transit time, and enteropooling models. Dose selection was made based on the results from acute oral toxicity test as well as pilot study using 8 experimental animals. Doses of 100, 200, and 400 mg/kg were designated as low, moderate, and high dose, respectively, in the present study. Stock solutions were prepared freshly on the day of experiment.

### 2.3. Determination of In Vivo Antidiarrheal Activity

#### 2.3.1. Castor Oil Induced Diarrhea Model

A method described by Maniyar et al. [24] was followed for the current study. In this experiment, Swiss albino mice of either sex were deprived of food for 18 h with free access to water and divided randomly into groups of six mice each. The negative control group received 10 mL/kg of distilled water (control for 80% MeOH-E) or 2% Tween 80 in distilled water (control for fraction groups) and the test groups received 100, 200, and 400 mg/kg body weight of 80% MeOH-E or solvent fractions while the positive control group was given loperamide 3 mg/kg orally. Separate control groups for 80% MeOH and fractions were arranged because of the difference in solubility between crude extracts and fractions. After 1 h of treatment with the vehicles or test drugs, diarrhea was induced by administration of 0.5 mL of castor oil orally to each mouse. The experimental animals were then placed individually in cages with a white paper lined floor to collect fecal matters. The transparent paper was changed every h for a total of 4 h. During the observation period of 4 h, onset of diarrhea (the time interval in minutes between administration of castor oil and appearance of the first diarrheal stool), number and weight of wet stools, and total number and weight of fecal output were recorded for individual mouse. Finally, percentages of diarrheal inhibition, weight of wet, and total fecal output were calculated using formulas described as follows [25, 26]:

(1) Percentage  of  inhibition  %=Mean  number  of  WFC−Mean  number  of  WFTMean  number  of  WFC×100,

where WFC is wet feces in control group and WFT is wet feces in test group.

(2) Percentage  of  wet  fecal  output  %=Mean  weight  of  wet  feces  of  each  treatment  groupMean  weight  of  wet  feces  of  control×100,Percentage  of  total  fecal  output  %=Mean  fecal  weight  of  each  treatment  groupMean  fecal  weight  of  control×100,

#### 2.3.2. Castor Oil Induced Gastrointestinal Motility Test

Gastrointestinal transit (motility) was investigated in mice using the method mentioned somewhere [27]. Before commencement of the experiment, mice were fasted for 18 h but allowed free access to water and randomly allocated into groups of six animals in each group to determine the effect of 80% MeOH-E and fractions on gastrointestinal transit of a marker meal. Mice received distilled water or 2% Tween 80 in distilled water (10 mL/kg), 80% MeOH-E or solvent fractions (100, 200, and 400 mg/kg), and standard drug (loperamide 3 mg/kg) orally. After an hour of dosing, all of the mice were challenged with 0.5 mL of castor oil perorally to induce diarrhea. Then, 1 mL of charcoal meal (5% activated charcoal suspension in distilled water) was given orally an hour after castor oil administration. Thirty minutes later each mouse was then humanly sacrificed by cervical dislocation and small intestine was immediately dissected out from pylorus to caecum and placed length wise on a white paper. Distance travelled by the charcoal meal from pylorus and total length of the intestine were measured. Peristaltic index (PI) expressed as percentage of the distance travelled by the charcoal meal relative to the total length of the small intestine as well as percentage of inhibition of movement as a function of the control was calculated using the following formulas:

(3) Peristaltic  index  PI=Mean  distance  travelled  by  charcoal  mealMean  length  of  small  intestine×100,Percentage  of  inhibition  %=Dc−DtDc×100,

where Dc is mean distance travelled by the charcoal in the control group and Dt is mean distance travelled by the charcoal in the test group.

#### 2.3.3. Castor Oil Induced Enteropooling Test

Effects of 80% MeOH-E and solvent fractions on intraluminal fluid accumulation were determined using a method described by Degu and colleagues [28]. Prior to the experiment, the animals of either sex were fasted for 18 h and randomly divided into groups consisting of six mice in each group; they were pretreated with distilled water or 2% Tween 80 in distilled water (10 mL/kg), extract or fractions (100, 200, and 400 mg/kg), and standard drug (loperamide 3 mg/kg) orally. After 1 h of treatment, 0.5 mL of castor oil was given and animals were sacrificed by cervical dislocation 1 h following castor oil administration. The abdomen of each animal was then opened; the small intestine was removed, tied with thread at pyloric end and ileocaecal junction. The dissected small intestine was weighed and intestinal contents were then collected by milking into a graduated tube and their volume was measured. Weight of the intestine after milking was taken and the difference between full and empty intestines was calculated. Finally, percentage of inhibitions of intestinal secretion (volume and weight) by the extract or fractions were estimated relative to the negative control using the following formulas:

(4) Percentage  of  inhibition  by  using  MVIC=MVICC−MVICTMVICC×100,

where MVICC is mean volume of intestinal content of control group and MVICT is mean volume of intestinal content of test group.

(5) Percentage  of  inhibition  by  using  MWIC=MWICC−MWICTMWICC×100,

where MWICC is mean weight of intestinal content of control group and MWICT is mean weight of intestinal content of test group.

#### 2.3.4. Estimation of In Vivo Antidiarrheal Index

The in vivo antidiarrheal index (ADI) for 80% MeOH-E and solvent fractions was determined by combining three parameters taken from the aforementioned models. It was then expressed according to the following formula [29]:

(6) In  vivo  antidiarrheal  index  ADI=Dfreq×Gmeq×Pfreq3,

where Dfreq is the delay in defecation time as percentage of negative control, Gmeq is the gut meal travel reduction as percentage of negative control, and Pfreq is the reduction in purging frequency in the number of wet stools as percentage of negative control.

#### 2.3.5. Preliminary Phytochemical Screening

The crude extract as well as chloroform, n-butanol, and aqueous fractions of* M. kummel *fruits was tested for the presence of various phytochemical classes of compounds such as anthraquinones, tannins, saponins, flavonoids, terpenoids, alkaloids, glycosides, steroids, and phenols using previously described methods [30–32].

#### 2.3.6. Acute Oral Toxicity Test

Acute oral toxicity test for 80% MeOH-E of* M. kummel *fruits was performed according to the Organization for Economic Cooperation and Development (OECD) guideline 425 [33]. Five female albino mice of 6–8 weeks were used for each test. All mice were fasted (food but not water) for 4 h before and 2 h after the administration of the extract. First, a sighting study was performed to determine the starting dose. For this, a single female mouse was given 2000 mg/kg of the extract as a single dose by oral gavage. Since no death was observed within 24 h, additional four mice were used and administered the same dose of the extract. The animals were housed separately in cages and observed continuously for 4 h in 30 min interval and then for 14 consecutive days with an interval of 24 h for the general signs and symptoms of toxicity, food and water intake, and mortality.

### 2.4. Data Analysis

Data were analyzed using Statistical Package for Social Sciences, version 20. Experimental results obtained from this study were expressed as mean ± CI_95_ (95% confidence interval). The statistical analysis of data was done using one-way analysis of variance followed by Tukey's post hoc test for multiple comparisons, which was used to compare results among groups. Differences were considered statistically significant if *p* values were less than 0.05. Linear regression analysis was applied to assess dose dependency nature of antidiarrheal effect.

## 3. Results

### 3.1. Effect of* M. kummel *Fruits on Castor Oil Induced Diarrheal Model

In the course of observation for 4 h after castor oil administration, as presented in Table 1, the 80% MeOH-E of the fruits of* M. kummel* significantly delayed the onset of diarrhea and reduced the number and weight of wet and total stools at doses of 100 mg/kg (*p* < 0.05), 200 mg/kg (*p* < 0.05), and 400 mg/kg (*p* < 0.05) as compared with the negative control. Besides, the data revealed that percentage of diarrheal inhibitions were 71.40% (*p* < 0.05), 76.70% (*p* < 0.05), and 85.70% (*p* < 0.05) at the doses of 100, 200, and 400 mg/kg, respectively. The maximum dose of this extract (400 mg/kg) produced the maximum percentage of inhibition of defecation and the lowest percentage of mean fecal output when compared with the tested doses of the extract and positive control.

Data from the experiment revealed that percentage of diarrheal inhibitions obtained as compared with control were 60.82% (*p* < 0.05), 68.59% (*p* < 0.05), and 70.59% (*p* < 0.05) at the doses of 100, 200, and 400 mg/kg aqueous fractions, respectively. The aqueous fraction also showed a significant reduction in weight of both wet and total fecal output at 100 mg/kg (*p* < 0.05), 200 mg/kg (*p* < 0.05), and 400 mg/kg (*p* < 0.05) when compared with the negative control. Similarly, the n-butanol fraction significantly decreased the frequency and weight of wet and total feces at doses of 200 mg/kg (*p* < 0.05) and 400 mg/kg (*p* < 0.05), with the highest percentage of diarrheal inhibition (56.82%, *p* < 0.05) obtained at the latter dose of this fraction compared with the negative control. The chloroform fraction had significant antidiarrheal activity on castor oil induced diarrhea as compared with the negative control (*p* < 0.05), with lower value at 100 mg/kg and 200 mg/kg doses compared with the other two fractions. In addition, 100 mg/kg and 200 mg/kg chloroform fraction did not have any significant effect on weight of wet and total fecal outputs.

As depicted in Table 1, there was a dose-dependent reduction in the percentage of weight of wet and total fecal outputs in 80% MeOH-E (*R*^2^ = 1.000; *R*^2^ = 0.997, *p* < 0.05), chloroform fraction (*R*^2^ = 0.939; *R*^2^ = 0.992, *p* < 0.05), and n-butanol fraction (*R*^2^ = 0.984; *R*^2^ = 0.974, *p* < 0.05), respectively, with 400 mg/kg of the 80% MeOH-E displaying the highest effect (15.22% and 16.33%). As compared with the standard drug (28.26% and 34.69%), the 80% MeOH-E at its 400 mg/kg revealed the greatest impact on the percentage of fecal output.

### 3.2. Effect of* M. kummel *Fruits on Castor Oil Induced Gastrointestinal Motility

As presented in Table 2, the 80% MeOH-E significantly inhibited the intestinal transit of charcoal meal at all tested doses. The percentage of reduction of gastrointestinal transit of charcoal was 57.66% (*p* < 0.05), 63.14% (*p* < 0.05), and 68.98% (*p* < 0.05) at doses of 100, 200, and 400 mg/kg, respectively. The activity of 80% MeOH-E at 400 mg/kg was comparable to that of the standard drug, loperamide (66.06% at the dose of 3 mg/kg).

All the three fractions of fruits of* M. kummel *significantly inhibited gastrointestinal motility of charcoal meal at all tested doses as compared with vehicle treated group. The maximum effect was achieved by the aqueous fraction at 400 mg/kg with the charcoal meal traversing 56.45% of the total length of the small intestine (Table 2).

### 3.3. Effect of* M. kummel* Fruits on Castor Oil Induced Intestinal Secretion

In the gastrointestinal enteropooling test, 80% MeOH-E of* M. kummel* fruits showed significant reduction in both average volume (except the low dose, 100 mg/kg) and weight of intestinal contents at all tested doses as compared with the negative control (*p* < 0.05) (Table 3).

The aqueous and n-butanol fractions reduced the volume and weight of the intestinal contents significantly at all tested doses. Maximum percentage of inhibition of the volume of intestinal contents was observed at 400 mg/kg, being 56.32% (*p* < 0.05) and 57.47% (*p* < 0.05) for aqueous and n-butanol fractions, respectively. The chloroform fraction was devoid of any significant inhibitory effect on the volume and weight of intestinal contents up to 200 mg/kg as compared with the negative control (Table 3).

### 3.4. Effect of* M. kummel *Fruits on In Vivo Antidiarrheal Index

Estimates of in vivo ADI revealed that the greatest value was achieved at the dose of 400 mg/kg of 80% MeOH-E (96.00%) which is comparable to the standard drug, loperamide at the dose of 3 mg/kg (98.12%). Among all solvent fractions, aqueous fraction showed the highest in vivo ADI (80.33%) at the dose 400 mg/kg. Both 80% MeOH-E and solvent fractions showed dose-dependent increment in ADI value: 80% MeOH-E (*R*^2^ = 0.913), chloroform fraction (*R*^2^ = 0.887), n-butanol fraction (*R*^2^ = 0.941), and aqueous fraction (*R*^2^ = 0.832) (Table 4).

### 3.5. Preliminary Phytochemical Screening Results

Preliminary phytochemical screening of the 80% MeOH-E of* M. kummel* fruits revealed the presence of alkaloids, saponins, tannins, phenols, terpenoids, and flavonoids. Steroids, anthraquinones, and glycosides were absent in 80% MeOH-E of the plant. Among the solvent fractions, only the aqueous fraction tested positive for flavonoids. On the other hand, saponins and tannins were detected in both aqueous and n-butanol fractions. Phenols were common across all solvent fractions. Terpenoids were observed in both chloroform and n-butanol fractions. Among the three fractions, the aqueous fraction appeared to be qualitatively rich in secondary metabolites as shown from Table 5.

### 3.6. Acute Oral Toxicity Test

Eighty percent of methanol extract of the fruits of* M. kummel* was studied for oral acute toxicity at dose of 2000 mg/kg by oral route. The extract produced no mortality and any apparent signs of toxicity when observed for first 4 h with 30 min interval and followed by daily observations for 14 days following oral administration of a single dose of 2000 mg/kg. In addition, neither food nor water intake was found to be reduced during the period of 14 days. The absence of mortality and signs of overt toxicity up to five times the maximum effective dose of the extract used in the experiment suggested that 80% MeOH-E has a wider safety margin and median lethal dose (LD_50_) value is greater than 2000 mg/kg in mice.

## 4. Discussion

People customarily use plant(s) or plant-derived preparations considering them to be efficacious against diarrheal disorders without any scientific basis to explain the action of such plants [34]. The aim of the present study was to experimentally evaluate the folkloric acclaimed use of* M. kummel* fruits, which are regarded to confer protection in diarrhea in Ethiopian traditional medicine. Solvent fractions and 80% MeOH-E of* M. kummel* fruits were substantiated with scientific evidence on the possible mode of action for their antidiarrheal activities.

In Ethiopia, fruits of* M. kummel* are consumed in the traditional medical practice of gastrointestinal abnormalities [15]. In the present study, authors selected 80% methanol to extract the fruits for secondary metabolites efficiently. The solvent fractions were employed to differentiate the predominant types of secondary metabolites with a better pharmacological activity [35].

In the current study, there was a statistically significant (*p* < 0.05) reduction in number and weight of fecal output and delayed onset of diarrhea in the test groups. The 80% MeOH-E tested at 100 mg/kg, 200 mg/kg, and 400 mg/kg significantly inhibited the frequency of defecation droppings when compared with untreated control mice (*p* < 0.05). The significant reduction in frequency of defecation, weight of wet stools, and weight of total stools signifies the efficacy of 80% MeOH-E of* M. kummel* fruits as antidiarrheal agent. This result is in support of previous claims in respect of antidiarrheal medicinal plants.

The aqueous, n-butanol, and chloroform fractions produced antidiarrheal effects in all parameters in castor oil induced diarrheal model, with the aqueous fraction being the most active fraction. In addition, both aqueous and n-butanol fractions significantly decreased the number of wet and total feces and weight of both wet and total stooling at 200 mg/kg as compared with the negative control (*p* < 0.05). The chloroform fraction significantly decreased the number of wet and total feces but not weight of both wet and total stooling at 200 mg/kg dose. Its low dose, 100 mg/kg, had significant effect in altering only the onset of diarrhea when compared with the negative control (*p* < 0.05). The insignificant activity of the chloroform fraction for some parameters at the low dose might be due to the inability of secondary metabolites to reach sufficient concentration. This argument is supported by the fact that activity would be apparent with increasing dose of the fraction. This could possibly suggest the localization of the active ingredients in the aqueous and n-butanol fractions. Moreover, it is plausible to assume that more polar secondary metabolites could be responsible for impact on the diarrheal parameters (onset of diarrhea, number of wet stools, total number of stools, weight of wet stools, and weight of total stools). This study was in line with other studies in which the aqueous and n-butanol fractions of different plants reduced the number and weight of stooling [36–38]. Generally, the fractions showed differences in potency in castor oil induced diarrheal model in the rank order of aqueous fraction > n-butanol fraction > chloroform fraction in all parameters. The difference in rank order of potency could emanate from the differential distribution of the secondary metabolites as depicted in Table 5.

In the present study, it was shown that the 80% MeOH-E significantly suppresses the propulsion of charcoal marker at all tested doses as compared with negative control. The percentage of inhibition of charcoal marker at 400 mg/kg dose (68.98%) of this extract was observed to be almost comparable to the standard drug (66.06% at the dose of 3 mg/kg). This finding suggests that the extract has ability to influence the peristaltic movement of intestine thereby indicating presence of an intestinal antimotility activity. This decrease in motility facilitates the absorption of electrolytes and then water [39], which might be responsible for the decrease in the number and weight of wet feces observed with the extract. Both aqueous and n-butanol fractions had statistically significant antispasmodic effects with the highest effect revealed at 400 mg/kg of aqueous fraction (56.45%, *p* < 0.05). Even though the chloroform fraction had a significant effect on the charcoal meal test, it failed to show a significant effect on number and weight of wet feces, particularly at the low dose. This could be explained by the fact that antimotility effect might not be a necessary and sufficient factor for counteracting diarrhea and it should be supplemented with a certain degree of antisecretory activity.

In the present study, enteropooling model was employed to assess the impact of test fractions and extract on secretory components of diarrhea. In this model, the 80% MeOH-E, aqueous, and n-butanol fractions significantly reduced the average volume and weight of intestinal contents at all tested doses when compared with the negative control group (*p* < 0.05). These findings support the evidence for the extract and fractions' antidiarrheal action to be mediated by antisecretory mechanism. On the contrary, the chloroform fraction had a significant inhibition of intestinal fluid accumulation only at the high dose (400 mg/kg). This further supports the significant activity of these fractions on the number and weight of wet feces on the castor oil induced diarrheal model, which is in agreement with other studies by which the antienteropooling effects of the extract are related to its antidiarrheal effect [40]. In fact, the ability of chloroform fraction to reduce the volume and weight of intestinal contents only at 400 mg/kg (*p* < 0.05) lends further support to the limited antidiarrheal effects in the castor oil induced diarrheal model. The significant inhibition of the castor oil induced enteropooling by aqueous fraction in mice suggests that the extract probably produces relief in diarrhea through its spasmolytic and antienteropooling effects. These findings are in consonance with the observations reported for aqueous leaf extract of* Byrsocarpus coccineus *(Connaraceae) [41] and for aqueous whole plant extract of* Mezoneuron benthamianum* (Caesalpiniaceae) [42]. This may promote reabsorption of materials in the intestine due to decrease propulsion of material in the intestinal tract.

The in vivo ADI is a measure of the combined effects of three parameters such as delay in onset of diarrheal stools, intestinal motility, and purging frequency in number of wet stools [43, 44]. Generally, high ADI value indicates a measure of how much effective an extract is in treating diarrhea [45, 46]. The highest in vivo ADI value was produced by the 80% MeOH-E at its high dose which is directly related to its antidiarrheal activity in all of the three models. This reinforces the notion that the extract is endowed with best antidiarrheal activity compared with solvent fractions. Moreover, the aqueous fraction showed the highest ADI value at its maximum dose as compared with the other fractions. Conversely, the chloroform fraction, which had little antidiarrheal activity in all models, exhibited the lowest ADI, pointing to the fact that ADI is a useful parameter in ranking antidiarrheal agents.

In the present investigation, the acute toxicity profile of the fruits of* M. kummel* was determined based on OECD guideline 425 [33], which recommends the use of minimal number of experimental animals. The LD_50_ for the 80% MeOH-E was found to be >2000 mg/kg. Generally, if the LD_50_ value of the test chemical is more than 3-fold of the minimum effective dose, the test substance can therefore be categorized under experimentally safe substances [47]. Since the 80% MeOH-E had LD_50_ value of more than three times of the minimum effective dose (100 mg/kg), it can be taken as a good candidate for further studies. According to the World Health Organization hazard classification systems based on LD_50_, the 80% MeOH-E of the fruits of* M. kummel *with LD_50_ > 2000 mg/kg might be designated as “slightly hazardous” or “unlikely to present acute hazard” taking into consideration of the species similarity between rats and mice [48].

## 5. Conclusion

The results of the present study revealed that 80% MeOH-E of* M. kummel* fruits is endowed with a promising antidiarrheal activity. Moreover, all the three fractions possessed varying degree of antidiarrheal activity, with the aqueous fraction being the most active followed by the n-butanol fraction and then chloroform fraction in all the three models used in the current study. These findings provide scientific evidence for the next lead compound discovery and development undertakings from* M. kummel* fruits, which have multiple modes of action of antidiarrheal action.

## Figures and Tables

**Table 1:** Antidiarrheal effects of 80% MeOH-E and solvent fractions of *M. kummel* fruits on castor oil induced diarrheal model in mice.

Extracts	Dose administered	Onset of diarrhea (min)	Number of wet feces	Total number of feces	Average weight of wet feces (gm)	Average weight of total feces (gm)	% reduction	% wet fecal outputs	% total fecal outputs
80% MeOH-E	Control^1^	44.33 ± 1.65	10.50 ± 1.18	11.83 ± 0.94	0.46 ± 0.11	0.49 ± 0.12	—	—	—
	100 mg/kg	67.00 ± 1.13^*∗*†‡*₸*^	3.00 ± 0.72^*∗*^	4.17 ± 1.55^*∗*^	0.16 ± 0.06^*∗*^	0.21 ± 0.05^*∗*^	71.40	34.78	42.86
	200 mg/kg	93.33 ± 1.63^*∗*†*₸*^	2.83 ± 0.94^*∗*^	3.50 ± 0.67^*∗*^	0.13 ± 0.03^*∗*^	0.17 ± 0.03^*∗*^	76.70	28.26	34.69
	400 mg/kg	110.67 ± 1.63^*∗*†^	1.50 ± 0.84^*∗*^	2.33 ± 0.83^*∗*^	0.07 ± 0.03^*∗*^	0.08 ± 0.04^*∗*^	85.70	15.22	16.33
	3 mg/kg loperamide	124.17 ± 1.09^*∗*^	2.17 ± 0.60^*∗*^	2.67 ± 0.65^*∗*^	0.13 ± 0.02^*∗*^	0.17 ± 0.02^*∗*^	79.40	28.26	34.69

Solvent fractions	Control^2^	53.17 ± 2.05	8.50 ± 0.84	9.67 ± 1.10	0.41 ± 0.02	0.45 ± 0.03	—	—	—

Solvent fractions	CF100 mg/kg	72.00 ± 2.02^*∗*†‡*₸*^	7.67 ± 1.21^†*₸*^	9.33 ± 0.97^†*₸*^	0.40 ± 0.10^†*₸*^	0.44 ± 0.09^†*₸*^	9.76	97.56	97.78
	CF200 mg/kg	86.50 ± 1.50^*∗*†*₸*^	5.83 ± 0.94^*∗*†^	7.50 ± 0.98^*∗*†^	0.30 ± 0.06^†^	0.37 ± 0.06^†^	31.41	73.17	82.22
	CF400 mg/kg	101.17 ± 2.74^*∗*†^	5.17 ± 0.94^*∗*†^	7.17 ± 0.60^*∗*†^	0.22 ± 0.06^*∗*^	0.27 ± 0.05^*∗*^	39.18	53.66	60.00

Solvent fractions	n-BF100 mg/kg	88.33 ± 1.73^*∗*†‡*₸*^	6.83 ± 1.18^†*₸*^	7.33 ± 0.97^*∗*†^	0.32 ± 0.03^*∗*†*₸*^	0.33 ± 0.03^*∗*†*₸*^	19.65	78.05	73.33
	n-BF200 mg/kg	102.17 ± 2.1^*∗*†*₸*^	4.67 ± 1.30^*∗*†^	6.33 ± 0.82^*∗*†^	0.29 ± 0.02^*∗*†*₸*^	0.31 ± 0.01^*∗*†*₸*^	45.06	70.73	68.89
	n-BF400 mg/kg	123.17 ± 1.78^*∗*†^	3.67 ± 1.30^*∗*^	5.50 ± 0.84^*∗*†^	0.18 ± 0.02^*∗*†^	0.22 ± 0.02^*∗*†^	56.82	43.90	48.89

Solvent fractions	AF100 mg/kg	92.67 ± 1.40^*∗*†‡*₸*^	3.33 ± 0.66^*∗*^	3.83 ± 0.60^*∗*^	0.26 ± 0.02^*∗*†‡*₸*^	0.27 ± 0.02^*∗*†^	60.82	63.41	60.00
	AF200 mg/kg	113.83 ± 1.1^*∗*†*₸*^	2.67 ± 0.82^*∗*^	3.83 ± 0.94^*∗*^	0.18 ± 0.01^*∗*†^	0.22 ± 0.03^*∗*†^	68.59	43.90	48.89
	AF400 mg/kg	122.33 ± 0.97^*∗*†^	2.50 ± 1.10^*∗*^	3.67 ± 0.82^*∗*^	0.18 ± 0.02^*∗*†^	0.21 ± 0.02^*∗*^	70.59	43.90	46.67

Solvent fractions	3 mg/kg loperamide	134.83 ± 1.38^*∗*^	1.67 ± 0.66^*∗*^	2.67 ± 0.66^*∗*^	0.13 ± 0.02^*∗*^	0.16 ± 0.02^*∗*^	80.35	31.71	35.56

Values are expressed as mean ± CI_95_ (*n* = 6); analysis was performed using one-way ANOVA followed by Tukey's post hoc test; ^*∗*^compared with control values; ^†^compared with loperamide; ^‡^compared with 200 mg/kg; ^*₸*^compared with 400 mg/kg; ^*∗*†‡*₸*^*p* < 0.05; 80% MeOH-E = 80% methanolic extract; CF = chloroform fraction; n-BF = n-butanol fraction; AF = aqueous fraction; CI_95_ = 95% confidence interval. Controls are 10 mL/kg distilled water (^1^for 80% MeOH-E) and 2% Tween 80 in distilled water (^2^for chloroform, n-butanol, and aqueous fractions).

**Table 2:** Effects of 80% MeOH-E and solvent fractions of *M. kummel* fruits on castor oil induced gastrointestinal motility in mice.

Extracts	Dose administered	Length of small intestine (cm)	Distance moved by the charcoal meal (cm)	Peristaltic index (%)	% inhibition
80% MeOH-E	Control^1^	56.83 ± 0.79	45.67 ± 0.97	80.36 ± 1.76	—
	100 mg/kg	52.50 ± 1.10	19.33 ± 0.97^*∗*†‡*₸*^	36.81 ± 1.39^*∗*†‡*₸*^	57.66
	200 mg/kg	53.67 ± 0.97	16.83 ± 0.94^*∗₸*^	31.35 ± 1.35^*∗₸*^	63.14
	400 mg/kg	57.50 ± 0.84	14.17 ± 1.18^*∗*^	24.64 ± 2.05^*∗*^	68.98
	3 mg/kg loperamide	58.67 ± 0.97	15.50 ± 0.84^*∗*^	26.42 ± 1.29^*∗*^	66.06

Solvent fractions	Control^2^	55.17 ± 1.38	47.83 ± 0.94	86.74 ± 1.76	—

Solvent fractions	CF100 mg/kg	53.67 ± 1.49	42.17 ± 1.71^*∗*†‡*₸*^	78.56 ± 2.02^*∗*†‡*₸*^	11.83
	CF200 mg/kg	54.83 ± 1.55	36.00 ± 1.43^*∗*†*₸*^	65.64 ± 1.50^*∗*†*₸*^	24.73
	CF400 mg/kg	54.67 ± 1.30	32.50 ± 1.50^*∗*†^	59.42 ± 1.55^*∗*†^	32.05

Solvent fractions	n-BF100 mg/kg	55.83 ± 0.94	38.67 ± 0.97^*∗*†‡*₸*^	69.31 ± 2.73^*∗*†‡*₸*^	19.15
	n-BF200 mg/kg	55.67 ± 1.40	33.00 ± 1.68^*∗*†*₸*^	59.24 ± 1.74^*∗*†*₸*^	31.06
	n-BF400 mg/kg	54.17 ± 1.18	27.67 ± 1.40^*∗*†^	51.05 ± 1.72^*∗*†^	42.20

Solvent fractions	AF100 mg/kg	55.17 ± 0.94	30.17 ± 1.18^*∗*†‡*₸*^	54.74 ± 2.90^*∗*†‡*₸*^	36.92
	AF200 mg/kg	55.83 ± 0.94	24.17 ± 0.94^*∗*†*₸*^	43.30 ± 1.92^*∗*†*₸*^	49.47
	AF400 mg/kg	55.00 ± 1.13	20.83 ± 1.18^*∗*†^	37.87 ± 1.90^*∗*†^	56.45

Solvent fractions	3 mg/kg loperamide	55.50 ± 0.84	17.33 ± 0.97^*∗*^	31.23 ± 1.66^*∗*^	63.77

Values are expressed as mean ± CI_95_ (*n* = 6); analysis was performed using one-way ANOVA followed by Tukey's post hoc test; ^*∗*^compared with control values; ^†^compared with loperamide; ^‡^compared with 200 mg/kg; ^*₸*^compared with 400 mg/kg; ^*∗*†‡*₸*^*p* < 0.05; 80% MeOH-E = 80% methanolic extract; CF = chloroform fraction; n-BF = n-butanol fraction; AF = aqueous fraction; CI_95_ = 95% confidence interval. Controls are 10 mL/kg distilled water (^1^for 80% MeOH-E) and 2% Tween 80 in distilled water (^2^for chloroform, n-butanol, and aqueous fractions).

**Table 3:** Effects of 80% MeOH-E and solvent fractions of the fruits of *M. kummel* on castor oil induced enteropooling in mice.

Extracts	Dose administered	Mean volume of small intestinal content (gm)	% inhibition	Mean weight of small intestinal content (mL)	% inhibition
80% MeOH-E	Control^1^	0.85 ± 0.08	—	1.21 ± 0.10	—
	100 mg/kg	0.63 ± 0.11	25.49	1.02 ± 0.17^*∗*†‡*₸*^	15.63
	200 mg/kg	0.52 ± 0.13^*∗*^	39.22	0.80 ± 0.02^*∗*^	33.61
	400 mg/kg	0.42 ± 0.09^*∗*^	50.98	0.69 ± 0.01^*∗*^	43.02
	3 mg/kg loperamide	0.45 ± 0.11^*∗*^	47.06	0.71 ± 0.01^*∗*^	41.36

Solvent fractions	Control^2^	0.87 ± 0.10	—	0.99 ± 0.10	—

Solvent fractions	CF100 mg/kg	0.75 ± 0.11^†*₸*^	13.79	0.83 ± 0.07^†^	16.16
	CF200 mg/kg	0.57 ± 0.10^†^	34.48	0.72 ± 0.10^†^	27.30
	CF400 mg/kg	0.50 ± 0.11^*∗*^	42.53	0.67 ± 0.10^*∗*†^	32.33

Solvent fractions	n-BF100 mg/kg	0.63 ± 0.11^*∗*†*₸*^	27.59	0.70 ± 0.10^*∗*†*₸*^	29.31
	n-BF200 mg/kg	0.58 ± 0.12^*∗*†^	33.32	0.60 ± 0.02^*∗*†^	39.40
	n-BF400 mg/kg	0.37 ± 0.11^*∗*^	57.47	0.53 ± 0.08^*∗*^	46.45

Solvent fractions	AF100 mg/kg	0.53 ± 0.08^*∗*†^	39.08	0.63 ± 0.02^*∗*†*₸*^	36.35
	AF200 mg/kg	0.42 ± 0.10^*∗*^	51.72	0.54 ± 0.02^*∗*†^	45.50
	AF400 mg/kg	0.38 ± 0.12^*∗*^	56.32	0.45 ± 0.03^*∗*^	54.53

Solvent fractions	3 mg/kg loperamide	0.30 ± 0.07^*∗*^	60.91	0.42 ± 0.04^*∗*^	57.58

Values are expressed as mean ± CI_95_ (*n* = 6); analysis was performed using one-way ANOVA followed by Tukey's post hoc test; ^*∗*^compared with control values; ^†^compared with loperamide; ^‡^compared with 200 mg/kg; ^*₸*^compared with 400 mg/kg; ^*∗*†‡*₸*^*p* < 0.05; 80% MeOH-E = 80% methanolic extract; CF = chloroform fraction; n-BF = n-butanol fraction; AF = aqueous fraction; CI_95_ = 95% confidence interval. Controls are 10 mL/kg distilled water (^1^for 80% MeOH-E) and 2% Tween 80 in distilled water (^2^for chloroform, n-butanol, and aqueous fractions).

**Table 4:** In vivo antidiarrheal indices of 80% MeOH-E and solvent fractions of *M. kummel* fruits.

Extracts	Dose administered	Delay in defecation (time of onset in min, Dfreq) (%)	Gut meal travel distance (Gmeq) (%)	Purging frequency in number of wet stools (Pfreq) (%)	Antidiarrheal index (ADI)
80% MeOHE	100 mg/kg	51.14	57.66	71.40	59.49
	200 mg/kg	110.53	63.14	76.70	81.19
	400 mg/kg	149.65	68.98	85.70	96.00
	3 mg/kg loperamide	180.10	66.06	79.40	98.12

Solvent fractions	CF100 mg/kg	35.41	11.83	9.76	15.99
	CF200 mg/kg	62.69	24.73	31.41	36.52
	CF400 mg/kg	90.28	32.05	39.18	48.40

Solvent fractions	n-BF100 mg/kg	66.13	19.15	19.65	29.20
	n-BF200 mg/kg	92.16	31.06	45.06	50.53
	n-BF400 mg/kg	131.65	42.20	56.82	68.09

Solvent fractions	AF100 mg/kg	74.29	36.92	60.82	55.05
	AF200 mg/kg	114.09	49.47	68.59	72.88
	AF400 mg/kg	130.07	56.45	70.59	80.33

Solvent fractions	3 mg/kg loperamide	153.58	63.77	80.35	92.32

80% MeOH-E = 80% methanolic extract, CF = chloroform fraction, n-BF = n-butanol fraction, and AF = aqueous fraction.

**Table 5:** Phytochemicals in 80% MeOH-E and solvent fractions of *M. kummel* fruits.

Secondary metabolites	Crude extract	Solvent fraction		
	80% MeOH-E	Chloroform fraction	n-Butanol fraction	Aqueous fraction
Saponins	+	−	+	+
Tannins	+	−	+	+
Steroids	−	−	−	−
Phenols	+	+	+	+
Flavonoids	+	−	−	+
Alkaloids	+	+	−	+
Terpenoids	+	+	+	−
Anthraquinones	−	−	−	−
Glycosides	−	−	−	−

80% MeOH-E = 80% methanol extract, + = present, and − = absent.

## Abbreviations

ADI: Antidiarrheal index
80% MeOH-E: 80% methanolic extract
GI: Gastrointestinal
LD_50_: Median lethal dose
OECD: Organization for Economic Cooperation and Development
WHO: World Health Organization.

## Data Access

Data is available at Addis Ababa University Institutional Repository in a form of graduate student thesis (http://etd.aau.edu.et/handle/123456789/11148).
